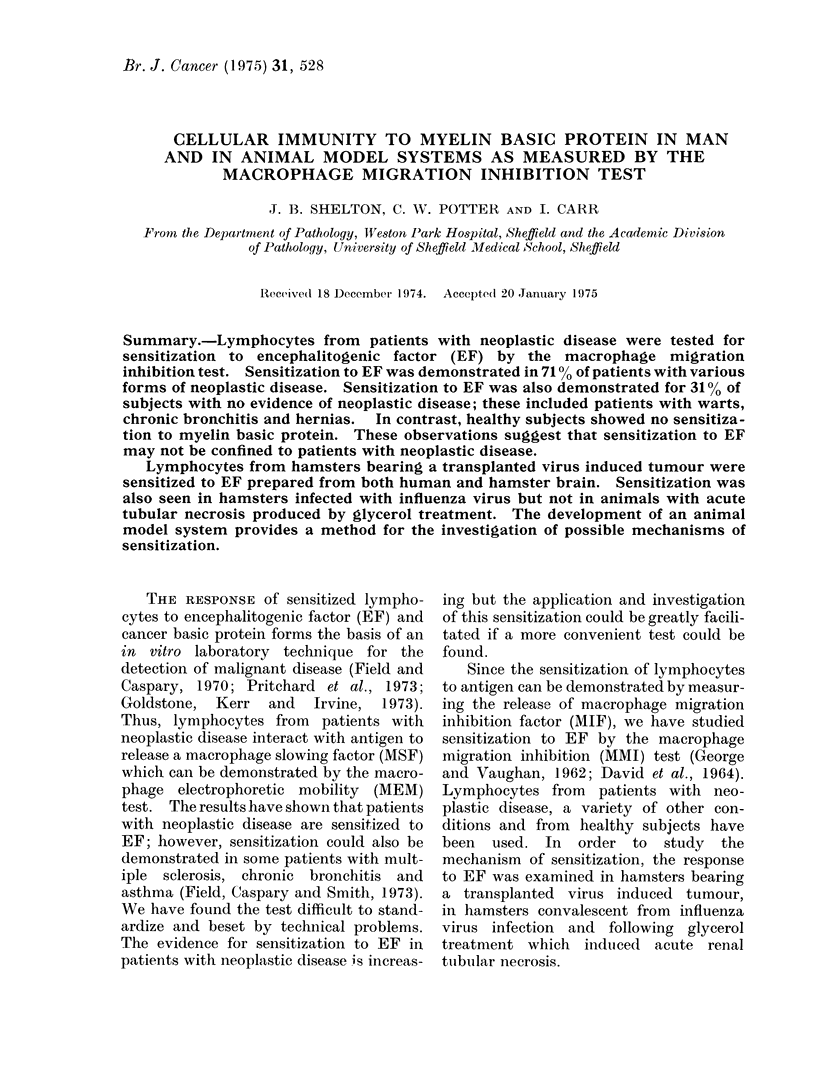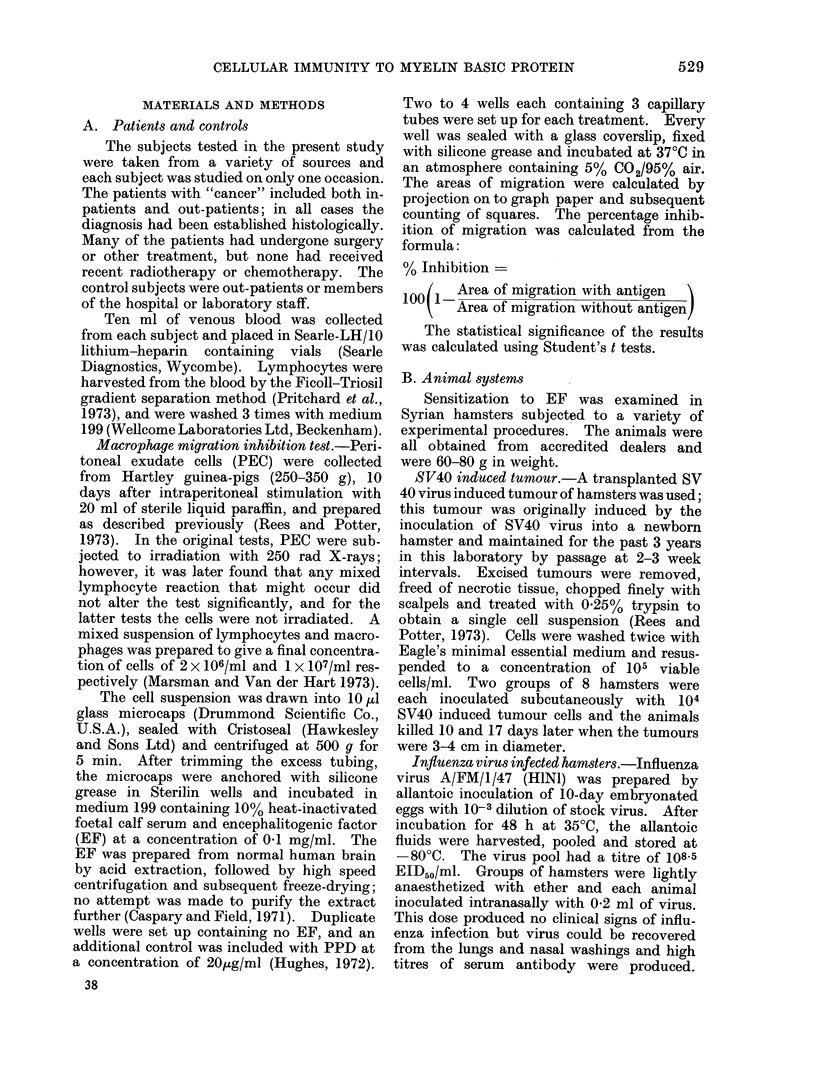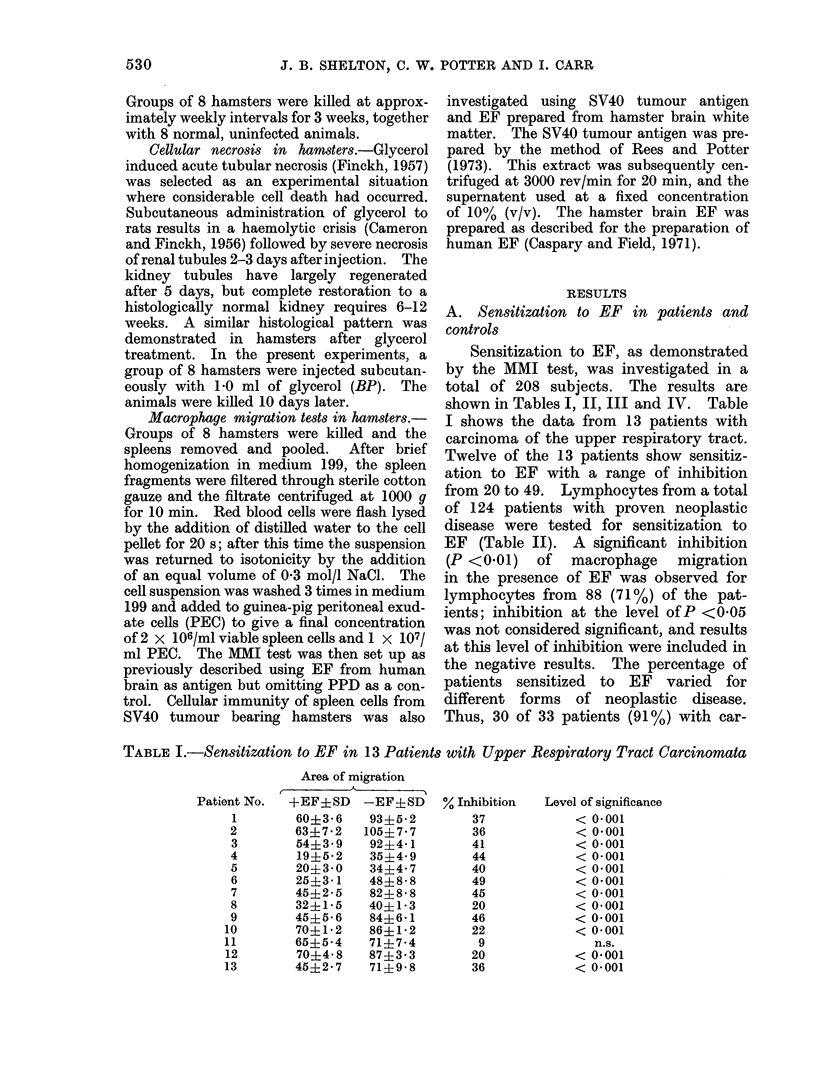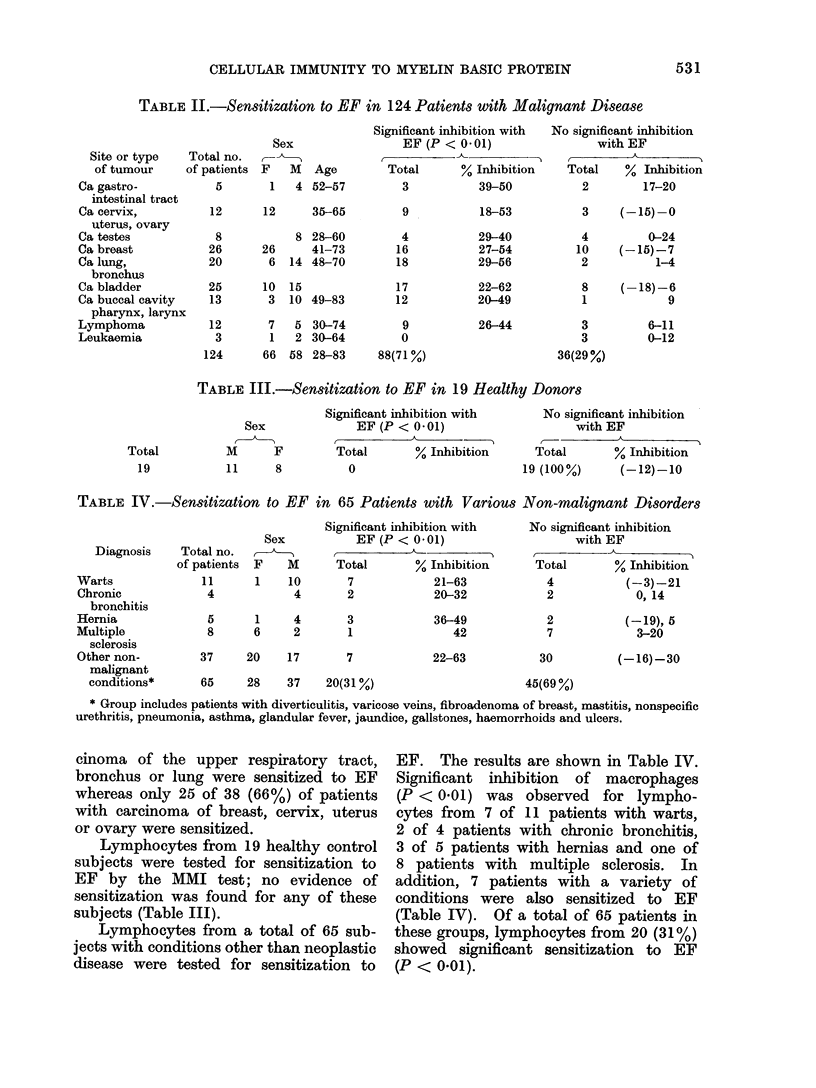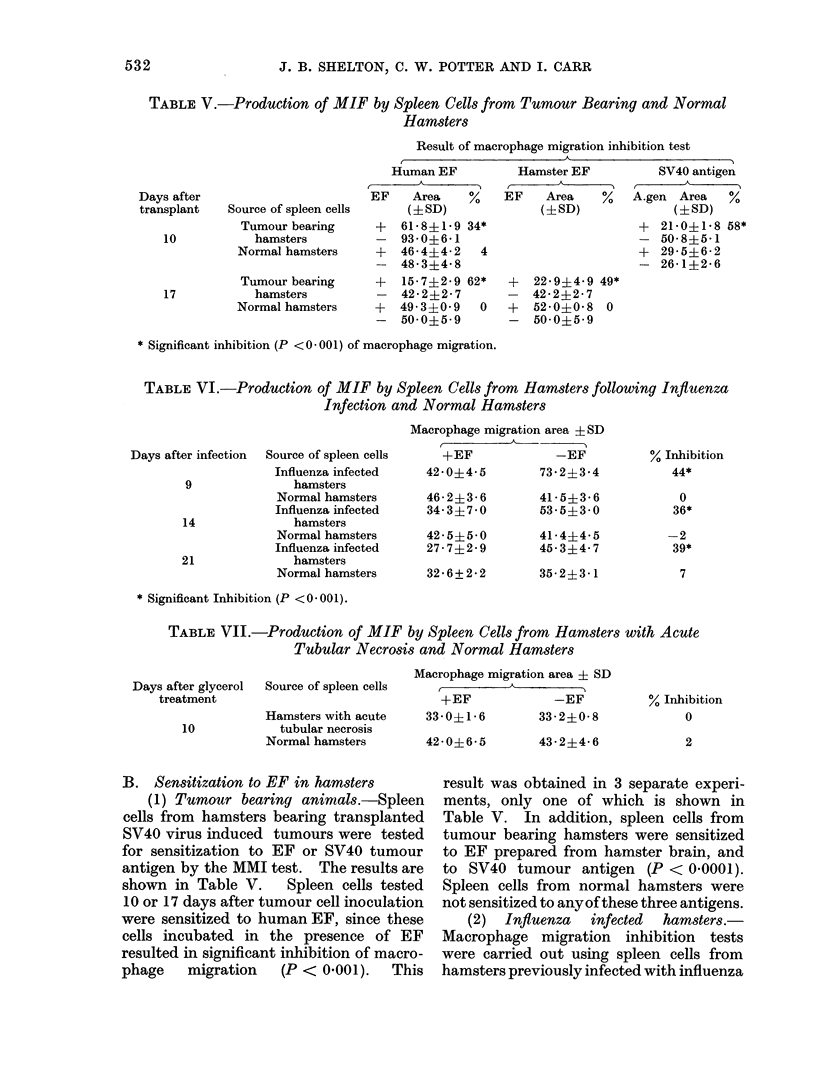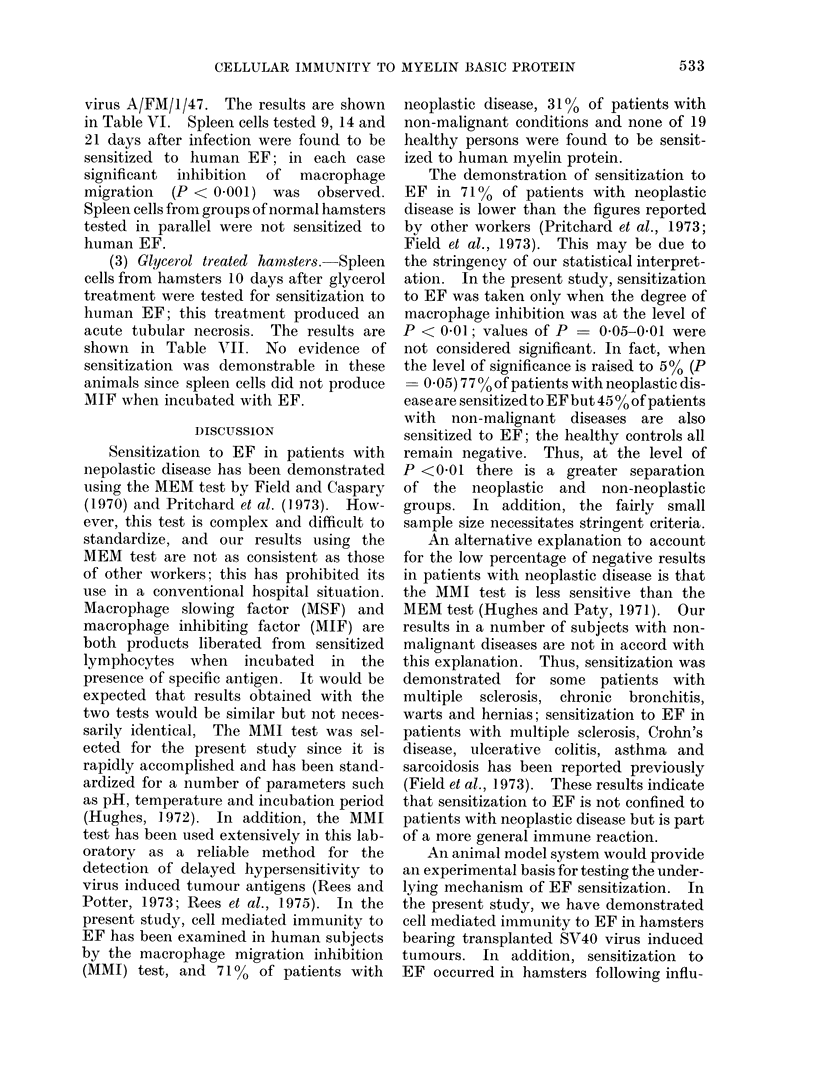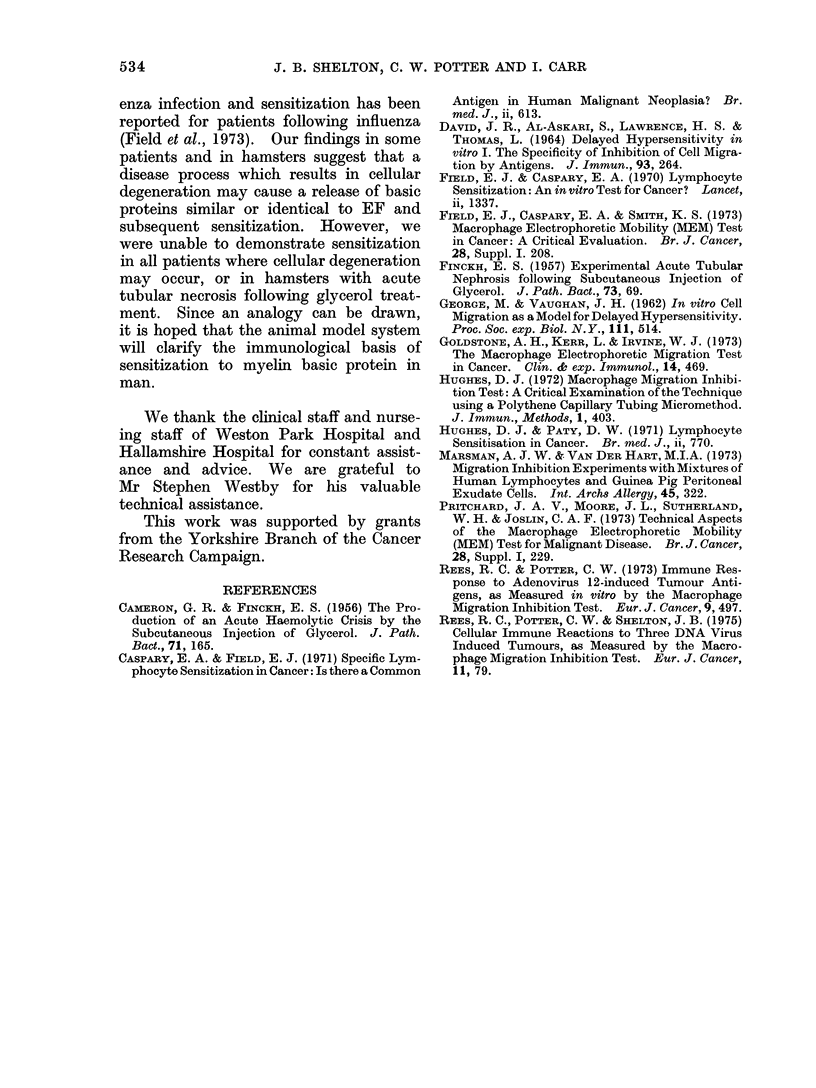# Cellular immunity to myelin basic protein in man and in animal model systems as measured by the macrophage migration inhibition test.

**DOI:** 10.1038/bjc.1975.93

**Published:** 1975-05

**Authors:** J. B. Shelton, C. W. Potter, I. Carr

## Abstract

Lymphocytes from patients with neoplastic disease were tested for sensitization to encephalitogenic factor (EF) by the macrophage migration inhibition test. Sensitization to EF was demonstrated in 71% of patients with various forms of neoplastic disease. Sensitization to EF was also demonstrated for 31% of subjects with no evidence of neoplastic disease; these included patients with warts, chronic bronchitis and hernias. In contrast, healthy subjects showed no sensitization to myelin basic protein. These observations suggest that sensitization to EF may not be confined to patients with neoplastic disease. Lymphocytes from hamsters bearing a transplanted virus induced tumour were sensitized to EF prepared from both human and hamster brain. Sensitization was also seen in hamsters infected with influenza virus but not in animals with acute tubular necrosis produced by glycerol treatment. The development of an animal model system provides a method for the investigation of possible mechanisms of sensitization.


Br. J. Cancer (1975) 31, 528

### CELLULAR IMMUNITY TO MYELIN BASIC PROTEIN IN MAN AND IN ANIMAL MODEL SYSTEMS AS MEASURED BY THE MACROPHAGE MIGRATION INHIBITION TEST

J. B. SHELTON, C. W. POTTER AND I. CARR

*From the Department of Pathology, Weston Park Hospital, Sheffield and the Academic Division of Pathology, University of Sheffield Medical School, Sheffield*

Received 18 December 1974. Accepted 20 January 1975

**Summary.—Lymphocytes from patients with neoplastic disease were tested for sensitization to encephalitogenic factor (EF) by the macrophage migration inhibition test. Sensitization to EF was demonstrated in 71% of patients with various forms of neoplastic disease. Sensitization to EF was also demonstrated for 31% of subjects with no evidence of neoplastic disease; these included patients with warts, chronic bronchitis and hernias. In contrast, healthy subjects showed no sensitization to myelin basic protein. These observations suggest that sensitization to EF may not be confined to patients with neoplastic disease.**

**Lymphocytes from hamsters bearing a transplanted virus induced tumour were sensitized to EF prepared from both human and hamster brain. Sensitization was also seen in hamsters infected with influenza virus but not in animals with acute tubular necrosis produced by glycerol treatment. The development of an animal model system provides a method for the investigation of possible mechanisms of sensitization.**

THE RESPONSE of sensitized lymphocytes to encephalitogenic factor (EF) and cancer basic protein forms the basis of an *in vitro* laboratory technique for the detection of malignant disease (Field and Caspary, 1970; Pritchard *et al.*, 1973; Goldstone, Kerr and Irvine, 1973). Thus, lymphocytes from patients with neoplastic disease interact with antigen to release a macrophage slowing factor (MSF) which can be demonstrated by the macrophage electrophoretic mobility (MEM) test. The results have shown that patients with neoplastic disease are sensitized to EF; however, sensitization could also be demonstrated in some patients with multiple sclerosis, chronic bronchitis and asthma (Field, Caspary and Smith, 1973). We have found the test difficult to standardize and beset by technical problems. The evidence for sensitization to EF in patients with neoplastic disease is increasing but the application and investigation of this sensitization could be greatly facilitated if a more convenient test could be found.

Since the sensitization of lymphocytes to antigen can be demonstrated by measuring the release of macrophage migration inhibition factor (MIF), we have studied sensitization to EF by the macrophage migration inhibition (MMI) test (George and Vaughan, 1962; David *et al.*, 1964). Lymphocytes from patients with neoplastic disease, a variety of other conditions and from healthy subjects have been used. In order to study the mechanism of sensitization, the response to EF was examined in hamsters bearing a transplanted virus induced tumour, in hamsters convalescent from influenza virus infection and following glycerol treatment which induced acute renal tubular necrosis.

### MATERIALS AND METHODS

### A. *Patients and controls*

The subjects tested in the present study were taken from a variety of sources and each subject was studied on only one occasion. The patients with "cancer" included both in-patients and out-patients; in all cases the diagnosis had been established histologically. Many of the patients had undergone surgery or other treatment, but none had received recent radiotherapy or chemotherapy. The control subjects were out-patients or members of the hospital or laboratory staff.

Ten ml of venous blood was collected from each subject and placed in Searle-LH/10 lithium–heparin containing vials (Searle Diagnostics, Wycombe). Lymphocytes were harvested from the blood by the Ficoll–Triosil gradient separation method (Pritchard *et al.*, 1973), and were washed 3 times with medium 199 (Wellcome Laboratories Ltd, Beckenham).

*Macrophage migration inhibition test.*—Peritoneal exudate cells (PEC) were collected from Hartley guinea-pigs (250–350 g), 10 days after intraperitoneal stimulation with 20 ml of sterile liquid paraffin, and prepared as described previously (Rees and Potter, 1973). In the original tests, PEC were subjected to irradiation with 250 rad X-rays; however, it was later found that any mixed lymphocyte reaction that might occur did not alter the test significantly, and for the latter tests the cells were not irradiated. A mixed suspension of lymphocytes and macrophages was prepared to give a final concentration of cells of $2\times10^6$/ml and $1\times10^7$/ml respectively (Marsman and Van der Hart 1973).

The cell suspension was drawn into 10 $\mu$l glass microcaps (Drummond Scientific Co., U.S.A.), sealed with Cristoseal (Hawkesley and Sons Ltd) and centrifuged at 500 *g* for 5 min. After trimming the excess tubing, the microcaps were anchored with silicone grease in Sterilin wells and incubated in medium 199 containing 10% heat-inactivated foetal calf serum and encephalitogenic factor (EF) at a concentration of 0·1 mg/ml. The EF was prepared from normal human brain by acid extraction, followed by high speed centrifugation and subsequent freeze-drying; no attempt was made to purify the extract further (Caspary and Field, 1971). Duplicate wells were set up containing no EF, and an additional control was included with PPD at a concentration of 20$\mu$g/ml (Hughes, 1972). Two to 4 wells each containing 3 capillary tubes were set up for each treatment. Every well was sealed with a glass coverslip, fixed with silicone grease and incubated at 37°C in an atmosphere containing 5% $CO_2$/95% air. The areas of migration were calculated by projection on to graph paper and subsequent counting of squares. The percentage inhibition of migration was calculated from the formula:

$$\% \text{ Inhibition} = 100\left(1-\frac{\text{Area of migration with antigen}}{\text{Area of migration without antigen}}\right)$$

The statistical significance of the results was calculated using Student's *t* tests.

### B. *Animal systems*

Sensitization to EF was examined in Syrian hamsters subjected to a variety of experimental procedures. The animals were all obtained from accredited dealers and were 60–80 g in weight.

*SV40 induced tumour.*—A transplanted SV 40 virus induced tumour of hamsters was used; this tumour was originally induced by the inoculation of SV40 virus into a newborn hamster and maintained for the past 3 years in this laboratory by passage at 2–3 week intervals. Excised tumours were removed, freed of necrotic tissue, chopped finely with scalpels and treated with 0·25% trypsin to obtain a single cell suspension (Rees and Potter, 1973). Cells were washed twice with Eagle's minimal essential medium and resuspended to a concentration of $10^5$ viable cells/ml. Two groups of 8 hamsters were each inoculated subcutaneously with $10^4$ SV40 induced tumour cells and the animals killed 10 and 17 days later when the tumours were 3–4 cm in diameter.

*Influenza virus infected hamsters.*—Influenza virus A/FM/1/47 (HlNl) was prepared by allantoic inoculation of 10-day embryonated eggs with $10^{-3}$ dilution of stock virus. After incubation for 48 h at 35°C, the allantoic fluids were harvested, pooled and stored at −80°C. The virus pool had a titre of $10^{8\cdot5}$ $EID_{50}$/ml. Groups of hamsters were lightly anaesthetized with ether and each animal inoculated intranasally with 0·2 ml of virus. This dose produced no clinical signs of influenza infection but virus could be recovered from the lungs and nasal washings and high titres of serum antibody were produced.

Groups of 8 hamsters were killed at approximately weekly intervals for 3 weeks, together with 8 normal, uninfected animals.

*Cellular necrosis in hamsters.*—Glycerol induced acute tubular necrosis (Finckh, 1957) was selected as an experimental situation where considerable cell death had occurred. Subcutaneous administration of glycerol to rats results in a haemolytic crisis (Cameron and Finckh, 1956) followed by severe necrosis of renal tubules 2–3 days after injection. The kidney tubules have largely regenerated after 5 days, but complete restoration to a histologically normal kidney requires 6–12 weeks. A similar histological pattern was demonstrated in hamsters after glycerol treatment. In the present experiments, a group of 8 hamsters were injected subcutaneously with 1·0 ml of glycerol (*BP*). The animals were killed 10 days later.

*Macrophage migration tests in hamsters.*—Groups of 8 hamsters were killed and the spleens removed and pooled. After brief homogenization in medium 199, the spleen fragments were filtered through sterile cotton gauze and the filtrate centrifuged at 1000 *g* for 10 min. Red blood cells were flash lysed by the addition of distilled water to the cell pellet for 20 s; after this time the suspension was returned to isotonicity by the addition of an equal volume of 0·3 mol/l NaCl. The cell suspension was washed 3 times in medium 199 and added to guinea-pig peritoneal exudate cells (PEC) to give a final concentration of $2 \times 10^6$/ml viable spleen cells and $1 \times 10^7$/ml PEC. The MMI test was then set up as previously described using EF from human brain as antigen but omitting PPD as a control. Cellular immunity of spleen cells from SV40 tumour bearing hamsters was also investigated using SV40 tumour antigen and EF prepared from hamster brain white matter. The SV40 tumour antigen was prepared by the method of Rees and Potter (1973). This extract was subsequently centrifuged at 3000 rev/min for 20 min, and the supernatent used at a fixed concentration of 10% (v/v). The hamster brain EF was prepared as described for the preparation of human EF (Caspary and Field, 1971).

### RESULTS

### A. *Sensitization to EF in patients and controls*

Sensitization to EF, as demonstrated by the MMI test, was investigated in a total of 208 subjects. The results are shown in Tables I, II, III and IV. Table I shows the data from 13 patients with carcinoma of the upper respiratory tract. Twelve of the 13 patients show sensitization to EF with a range of inhibition from 20 to 49. Lymphocytes from a total of 124 patients with proven neoplastic disease were tested for sensitization to EF (Table II). A significant inhibition ($P < 0·01$) of macrophage migration in the presence of EF was observed for lymphocytes from 88 (71%) of the patients; inhibition at the level of $P < 0·05$ was not considered significant, and results at this level of inhibition were included in the negative results. The percentage of patients sensitized to EF varied for different forms of neoplastic disease. Thus, 30 of 33 patients (91%) with car-

TABLE I.—*Sensitization to EF in* 13 *Patients with Upper Respiratory Tract Carcinomata*

| Patient No. | Area of migration: +EF±SD | Area of migration: −EF±SD | % Inhibition | Level of significance |
|---|---|---|---|---|
| 1 | 60±3·6 | 93±5·2 | 37 | < 0·001 |
| 2 | 63±7·2 | 105±7·7 | 36 | < 0·001 |
| 3 | 54±3·9 | 92±4·1 | 41 | < 0·001 |
| 4 | 19±5·2 | 35±4·9 | 44 | < 0·001 |
| 5 | 20±3·0 | 34±4·7 | 40 | < 0·001 |
| 6 | 25±3·1 | 48±8·8 | 49 | < 0·001 |
| 7 | 45±2·5 | 82±8·8 | 45 | < 0·001 |
| 8 | 32±1·5 | 40±1·3 | 20 | < 0·001 |
| 9 | 45±5·6 | 84±6·1 | 46 | < 0·001 |
| 10 | 70±1·2 | 86±1·2 | 22 | < 0·001 |
| 11 | 65±5·4 | 71±7·4 | 9 | n.s. |
| 12 | 70±4·8 | 87±3·3 | 20 | < 0·001 |
| 13 | 45±2·7 | 71±9·8 | 36 | < 0·001 |

TABLE II.—*Sensitization to EF in 124 Patients with Malignant Disease*

| Site or type of tumour | Total no. of patients | Sex F | Sex M | Age | Significant inhibition with EF ($P < 0\cdot01$) Total | % Inhibition | No significant inhibition with EF Total | % Inhibition |
|---|---|---|---|---|---|---|---|---|
| Ca gastro-intestinal tract | 5 | 1 | 4 | 52–57 | 3 | 39–50 | 2 | 17–20 |
| Ca cervix, uterus, ovary | 12 | 12 | | 35–65 | 9 | 18–53 | 3 | (−15)−0 |
| Ca testes | 8 | | 8 | 28–60 | 4 | 29–40 | 4 | 0–24 |
| Ca breast | 26 | 26 | | 41–73 | 16 | 27–54 | 10 | (−15)−7 |
| Ca lung, bronchus | 20 | 6 | 14 | 48–70 | 18 | 29–56 | 2 | 1–4 |
| Ca bladder | 25 | 10 | 15 | | 17 | 22–62 | 8 | (−18)−6 |
| Ca buccal cavity pharynx, larynx | 13 | 3 | 10 | 49–83 | 12 | 20–49 | 1 | 9 |
| Lymphoma | 12 | 7 | 5 | 30–74 | 9 | 26–44 | 3 | 6–11 |
| Leukaemia | 3 | 1 | 2 | 30–64 | 0 | | 3 | 0–12 |
| | 124 | 66 | 58 | 28–83 | 88(71%) | | 36(29%) | |

TABLE III.—*Sensitization to EF in 19 Healthy Donors*

| Total | Sex M | Sex F | Significant inhibition with EF ($P < 0\cdot01$) Total | % Inhibition | No significant inhibition with EF Total | % Inhibition |
|---|---|---|---|---|---|---|
| 19 | 11 | 8 | 0 | | 19 (100%) | (−12)−10 |

TABLE IV.—*Sensitization to EF in 65 Patients with Various Non-malignant Disorders*

| Diagnosis | Total no. of patients | Sex F | Sex M | Significant inhibition with EF ($P < 0\cdot01$) Total | % Inhibition | No significant inhibition with EF Total | % Inhibition |
|---|---|---|---|---|---|---|---|
| Warts | 11 | 1 | 10 | 7 | 21–63 | 4 | (−3)−21 |
| Chronic bronchitis | 4 | | 4 | 2 | 20–32 | 2 | 0, 14 |
| Hernia | 5 | 1 | 4 | 3 | 36–49 | 2 | (−19), 5 |
| Multiple sclerosis | 8 | 6 | 2 | 1 | 42 | 7 | 3–20 |
| Other non-malignant | 37 | 20 | 17 | 7 | 22–63 | 30 | (−16)−30 |
| conditions* | 65 | 28 | 37 | 20(31%) | | 45(69%) | |

* Group includes patients with diverticulitis, varicose veins, fibroadenoma of breast, mastitis, nonspecific urethritis, pneumonia, asthma, glandular fever, jaundice, gallstones, haemorrhoids and ulcers.

cinoma of the upper respiratory tract, bronchus or lung were sensitized to EF whereas only 25 of 38 (66%) of patients with carcinoma of breast, cervix, uterus or ovary were sensitized.

Lymphocytes from 19 healthy control subjects were tested for sensitization to EF by the MMI test; no evidence of sensitization was found for any of these subjects (Table III).

Lymphocytes from a total of 65 subjects with conditions other than neoplastic disease were tested for sensitization to EF. The results are shown in Table IV. Significant inhibition of macrophages ($P < 0\cdot01$) was observed for lymphocytes from 7 of 11 patients with warts, 2 of 4 patients with chronic bronchitis, 3 of 5 patients with hernias and one of 8 patients with multiple sclerosis. In addition, 7 patients with a variety of conditions were also sensitized to EF (Table IV). Of a total of 65 patients in these groups, lymphocytes from 20 (31%) showed significant sensitization to EF ($P < 0\cdot01$).

TABLE V.—*Production of MIF by Spleen Cells from Tumour Bearing and Normal Hamsters*

| Days after transplant | Source of spleen cells | Human EF: EF | Human EF: Area (±SD) | Human EF: % | Hamster EF: EF | Hamster EF: Area (±SD) | Hamster EF: % | SV40 antigen: A.gen | SV40 antigen: Area (±SD) | SV40 antigen: % |
|---|---|---|---|---|---|---|---|---|---|---|
| 10 | Tumour bearing hamsters | + | 61·8±1·9 | 34* | | | | + | 21·0±1·8 | 58* |
| | | − | 93·0±6·1 | | | | | − | 50·8±5·1 | |
| | Normal hamsters | + | 46·4±4·2 | 4 | | | | + | 29·5±6·2 | |
| | | − | 48·3±4·8 | | | | | − | 26·1±2·6 | |
| 17 | Tumour bearing hamsters | + | 15·7±2·9 | 62* | + | 22·9±4·9 | 49* | | | |
| | | − | 42·2±2·7 | | − | 42·2±2·7 | | | | |
| | Normal hamsters | + | 49·3±0·9 | 0 | + | 52·0±0·8 | 0 | | | |
| | | − | 50·0±5·9 | | − | 50·0±5·9 | | | | |

* Significant inhibition ($P < 0\cdot001$) of macrophage migration.

TABLE VI.—*Production of MIF by Spleen Cells from Hamsters following Influenza Infection and Normal Hamsters*

| Days after infection | Source of spleen cells | Macrophage migration area ±SD: +EF | −EF | % Inhibition |
|---|---|---|---|---|
| 9 | Influenza infected hamsters | 42·0±4·5 | 73·2±3·4 | 44* |
| | Normal hamsters | 46·2±3·6 | 41·5±3·6 | 0 |
| 14 | Influenza infected hamsters | 34·3±7·0 | 53·5±3·0 | 36* |
| | Normal hamsters | 42·5±5·0 | 41·4±4·5 | −2 |
| 21 | Influenza infected hamsters | 27·7±2·9 | 45·3±4·7 | 39* |
| | Normal hamsters | 32·6±2·2 | 35·2±3·1 | 7 |

* Significant Inhibition ($P < 0\cdot001$).

TABLE VII.—*Production of MIF by Spleen Cells from Hamsters with Acute Tubular Necrosis and Normal Hamsters*

| Days after glycerol treatment | Source of spleen cells | Macrophage migration area ± SD: +EF | −EF | % Inhibition |
|---|---|---|---|---|
| 10 | Hamsters with acute tubular necrosis | 33·0±1·6 | 33·2±0·8 | 0 |
| | Normal hamsters | 42·0±6·5 | 43·2±4·6 | 2 |

B. *Sensitization to EF in hamsters*

(1) *Tumour bearing animals.*—Spleen cells from hamsters bearing transplanted SV40 virus induced tumours were tested for sensitization to EF or SV40 tumour antigen by the MMI test. The results are shown in Table V. Spleen cells tested 10 or 17 days after tumour cell inoculation were sensitized to human EF, since these cells incubated in the presence of EF resulted in significant inhibition of macrophage migration ($P < 0\cdot001$). This result was obtained in 3 separate experiments, only one of which is shown in Table V. In addition, spleen cells from tumour bearing hamsters were sensitized to EF prepared from hamster brain, and to SV40 tumour antigen ($P < 0\cdot0001$). Spleen cells from normal hamsters were not sensitized to any of these three antigens.

(2) *Influenza infected hamsters.*—Macrophage migration inhibition tests were carried out using spleen cells from hamsters previously infected with influenza virus A/FM/1/47. The results are shown in Table VI. Spleen cells tested 9, 14 and 21 days after infection were found to be sensitized to human EF; in each case significant inhibition of macrophage migration ($P < 0 \cdot 001$) was observed. Spleen cells from groups of normal hamsters tested in parallel were not sensitized to human EF.

(3) *Glycerol treated hamsters.*—Spleen cells from hamsters 10 days after glycerol treatment were tested for sensitization to human EF; this treatment produced an acute tubular necrosis. The results are shown in Table VII. No evidence of sensitization was demonstrable in these animals since spleen cells did not produce MIF when incubated with EF.

### DISCUSSION

Sensitization to EF in patients with nepolastic disease has been demonstrated using the MEM test by Field and Caspary (1970) and Pritchard *et al.* (1973). However, this test is complex and difficult to standardize, and our results using the MEM test are not as consistent as those of other workers; this has prohibited its use in a conventional hospital situation. Macrophage slowing factor (MSF) and macrophage inhibiting factor (MIF) are both products liberated from sensitized lymphocytes when incubated in the presence of specific antigen. It would be expected that results obtained with the two tests would be similar but not necessarily identical, The MMI test was selected for the present study since it is rapidly accomplished and has been standardized for a number of parameters such as pH, temperature and incubation period (Hughes, 1972). In addition, the MMI test has been used extensively in this laboratory as a reliable method for the detection of delayed hypersensitivity to virus induced tumour antigens (Rees and Potter, 1973; Rees *et al.*, 1975). In the present study, cell mediated immunity to EF has been examined in human subjects by the macrophage migration inhibition (MMI) test, and 71% of patients with neoplastic disease, 31% of patients with non-malignant conditions and none of 19 healthy persons were found to be sensitized to human myelin protein.

The demonstration of sensitization to EF in 71% of patients with neoplastic disease is lower than the figures reported by other workers (Pritchard *et al.*, 1973; Field *et al.*, 1973). This may be due to the stringency of our statistical interpretation. In the present study, sensitization to EF was taken only when the degree of macrophage inhibition was at the level of $P < 0 \cdot 01$; values of $P = 0 \cdot 05–0 \cdot 01$ were not considered significant. In fact, when the level of significance is raised to 5% ($P = 0 \cdot 05$) 77% of patients with neoplastic disease are sensitized to EF but 45% of patients with non-malignant diseases are also sensitized to EF; the healthy controls all remain negative. Thus, at the level of $P < 0 \cdot 01$ there is a greater separation of the neoplastic and non-neoplastic groups. In addition, the fairly small sample size necessitates stringent criteria.

An alternative explanation to account for the low percentage of negative results in patients with neoplastic disease is that the MMI test is less sensitive than the MEM test (Hughes and Paty, 1971). Our results in a number of subjects with non-malignant diseases are not in accord with this explanation. Thus, sensitization was demonstrated for some patients with multiple sclerosis, chronic bronchitis, warts and hernias; sensitization to EF in patients with multiple sclerosis, Crohn's disease, ulcerative colitis, asthma and sarcoidosis has been reported previously (Field *et al.*, 1973). These results indicate that sensitization to EF is not confined to patients with neoplastic disease but is part of a more general immune reaction.

An animal model system would provide an experimental basis for testing the underlying mechanism of EF sensitization. In the present study, we have demonstrated cell mediated immunity to EF in hamsters bearing transplanted SV40 virus induced tumours. In addition, sensitization to EF occurred in hamsters following influenza infection and sensitization has been reported for patients following influenza (Field *et al.*, 1973). Our findings in some patients and in hamsters suggest that a disease process which results in cellular degeneration may cause a release of basic proteins similar or identical to EF and subsequent sensitization. However, we were unable to demonstrate sensitization in all patients where cellular degeneration may occur, or in hamsters with acute tubular necrosis following glycerol treatment. Since an analogy can be drawn, it is hoped that the animal model system will clarify the immunological basis of sensitization to myelin basic protein in man.

We thank the clinical staff and nursing staff of Weston Park Hospital and Hallamshire Hospital for constant assistance and advice. We are grateful to Mr Stephen Westby for his valuable technical assistance.

This work was supported by grants from the Yorkshire Branch of the Cancer Research Campaign.

### REFERENCES

Cameron, G. R. & Finckh, E. S. (1956) The Production of an Acute Haemolytic Crisis by the Subcutaneous Injection of Glycerol. *J. Path. Bact.*, **71**, 165.

Caspary, E. A. & Field, E. J. (1971) Specific Lymphocyte Sensitization in Cancer: Is there a Common Antigen in Human Malignant Neoplasia? *Br. med. J.*, ii, 613.

David, J. R., Al-Askari, S., Lawrence, H. S. & Thomas, L. (1964) Delayed Hypersensitivity *in vitro* I. The Specificity of Inhibition of Cell Migration by Antigens. *J. Immun.*, **93**, 264.

Field, E. J. & Caspary, E. A. (1970) Lymphocyte Sensitization: An *in vitro* Test for Cancer? *Lancet*, ii, 1337.

Field, E. J., Caspary, E. A. & Smith, K. S. (1973) Macrophage Electrophoretic Mobility (MEM) Test in Cancer: A Critical Evaluation. *Br. J. Cancer*, **28**, Suppl. I. 208.

Finckh, E. S. (1957) Experimental Acute Tubular Nephrosis following Subcutaneous Injection of Glycerol. *J. Path. Bact.*, **73**, 69.

George, M. & Vaughan, J. H. (1962) *In vitro* Cell Migration as a Model for Delayed Hypersensitivity. *Proc. Soc. exp. Biol. N.Y.*, **111**, 514.

Goldstone, A. H., Kerr, L. & Irvine, W. J. (1973) The Macrophage Electrophoretic Migration Test in Cancer. *Clin. & exp. Immunol.*, **14**, 469.

Hughes, D. J. (1972) Macrophage Migration Inhibition Test: A Critical Examination of the Technique using a Polythene Capillary Tubing Micromethod. *J. Immun., Methods*, **1**, 403.

Hughes, D. J. & Paty, D. W. (1971) Lymphocyte Sensitisation in Cancer. *Br. med. J.*, ii, 770.

Marsman, A. J. W. & Van Der Hart, M.I.A. (1973) Migration Inhibition Experiments with Mixtures of Human Lymphocytes and Guinea Pig Peritoneal Exudate Cells. *Int. Archs Allergy*, **45**, 322.

Pritchard, J. A. V., Moore, J. L., Sutherland, W. H. & Joslin, C. A. F. (1973) Technical Aspects of the Macrophage Electrophoretic Mobility (MEM) Test for Malignant Disease. *Br. J. Cancer*, **28**, Suppl. I, 229.

Rees, R. C. & Potter, C. W. (1973) Immune Response to Adenovirus 12-induced Tumour Antigens, as Measured *in vitro* by the Macrophage Migration Inhibition Test. *Eur. J. Cancer*, **9**, 497.

Rees, R. C., Potter, C. W. & Shelton, J. B. (1975) Cellular Immune Reactions to Three DNA Virus Induced Tumours, as Measured by the Macrophage Migration Inhibition Test. *Eur. J. Cancer*, **11**, 79.